# Workplace gaslighting: Conceptualization, development, and validation of a scale

**DOI:** 10.3389/fpsyg.2023.1099485

**Published:** 2023-03-30

**Authors:** Priyam Kukreja, Jatin Pandey

**Affiliations:** Organizational Behaviour and Human Resource Management Area, Indian Institute of Management Indore, Indore, India

**Keywords:** gaslighting at work, workplace abuse, supervisor-subordinate relationship, measurement, scale development

## Abstract

**Introduction:**

Gaslighting is a form of abuse that has transgressed the realms of romantic relationships to the relationships at work. Despite the growing literature on abuse at work, the conceptualization and measurement of gaslighting at work have received scarce attention. The study aimed to address this existing lacuna in the literature by conceptualizing and developing a measure of gaslighting at work.

**Methods:**

By drawing upon and integrating existing works of literature on harmful leader behaviors, workplace abuse, and workplace mistreatment, the authors have conceptualized the concept of gaslighting in a new context, i.e., work settings, and delineated its dimensions and conceptual boundaries. Among three different samples (total *N* = 679) of employees, the study developed a new 12-item measure of gaslighting in work relationships, the Gaslighting at Work Questionnaire (GWQ). The study further tested the psychometric properties of GWQ, namely, internal consistency, face, and construct validity of GWQ. Additionally, a time-lagged study was used to validate the scale within a nomological net of conceptual relationships.

**Results:**

Exploratory and confirmatory factor analysis supported a two-dimensional structure of gaslighting at work (trivialization and affliction). The psychometric properties of GWQ were established. Finally, using a time-lagged study, the scale was validated within a nomological net of conceptual relationships by showing the relationship of gaslighting at work with role conflict and job satisfaction.

**Discussion:**

The GWQ scale offers new opportunities to understand and measure gaslighting behaviors of a supervisor toward their subordinates in the work context. It adds to the existing literature on harmful leader behaviors, workplace abuse, and mistreatment by highlighting the importance of identifying and measuring gaslighting at work.

## 1. Introduction

The conversation around gaslighting has become increasingly popular and normalized in recent years. Gaslighting is a form of psychological abuse inflicted upon an individual, making the victim doubt his/her perceptions or capabilities (Gass and Nichols, 1988). Gaslighting has even been included in the purview of criminal domestic violence law of the United Kingdom in 2015 (Francis Hanna & Co, 2022). More than 300 people since then have been accused and charged for the same (Mikhailova, 2018). However, thus far, the scholarly literature has largely ignored this topic. While, a search for #gaslighting on Instagram returns over 600,000 results, less than 150 relevant scholarly articles exist in academic databases when a Google scholar search is conducted using the same phrase. Moreover, most of these scholarly articles focus on gaslighting in marriages (Gass and Nichols, 1988), closely knit friendships and intimate relationships (Miano et al., 2021). Some articles have also investigated gaslighting of kids by their parents (Riggs and Bartholomaeus, 2018). It should be noted that gaslighting may be perpetrated by any peer, family, spouse, or coworker and can be extremely damaging when the offender has a position of authority (Simon, 2011). Owing to the increasing pervasiveness of the term in employment relationships, it becomes of utmost importance to note that not enough attention has been paid so far on gaslighting in work settings, where a supervisor acts as an authority figure over the subordinate. What is more striking is that the existing literature on the topic is more qualitative in nature and lacks quantitative inquiry.

Despite previous research efforts, there is lack of consensus on the characterizing features of gaslighting and its subsequent definition. It serves as a great hindrance for conducting empirical studies. For instance, gaslighting has been defined as a “behavior in which one individual attempts to influence the judgment of a second individual by causing the latter to doubt the validity of his or her judgment” (Calef and Weinshel, 1981, p. 52), however, it lacks the specificity to enable gaslighting to be distinguished from other constructs in the same nomological space such as manipulation, brainwashing, and bullying.

Moreover, to boost research in this area, a reliable and valid measure of gaslighting is needed. First attempt to measure gaslighting, was made by Dr. Robin Stern in her book, The Gaslight Effect which displays a list of 20 statements on a dichotomous scale (Stern, 2007). However, it lacks methodological rigor like tests of validity, reliability, factor structure to name a few of the issues. Ever since there has been no measure focused on gaslighting occurring in work settings involving the dynamics of supervisor-subordinate. However, it is not possible to validate a measure of gaslighting, without a clear definition. Therefore, the purpose of the paper is to conceptualize gaslighting at work to support the development of its measure and further, create a psychometrically valid and reliable tool for measuring gaslighting behaviors in the power-laden relationship of supervisor and subordinate.

**Gaslighting:** The term ‘gaslighting’ was first used in a 1938 stage play named Gaslight, which was later, adapted into a 1944 film starring Ingrid Bergman and Charles Boyer. It was a tale of how a husband tricked his wife into believing she was crazy (Johnson et al., 2021). He caused the gas-powered lights in the home to flicker and denied it repeatedly when his wife complained. She endured emotional torment that was unfathomable. She eventually left her abusive marriage for a man who helped her reclaim her sense of self-worth by convincing her that what she believed to be true was not only her imagination.

Gaslighting is a continual process of sowing seeds of self-doubt in the mind of a person (Fielding-Singh and Dmowska, 2022). Where on one end, gaslighting can be a one-time incident, on the other hand, it can take the form of a sustained abuse (Johnson et al., 2021), and thus, the victim may not realize it in the early stages. Even the person who is gaslighting the other is usually not specifically aware to his/her behaviors specifically, how his/her behavior or actions are impacting the target. Some research has shown how the person indulging in such conduct has a narcissistic personality disorder (Boring, 2020) and continuously strives to attain dominance over the other person making him/her believe that whatever they feel is invalid (Boring, 2020). The importance of studying gaslighting behaviors can be studied from the knot theory of mind which states that abuse of the mind and emotions results in the construction of many knots of bad ideas and feelings, involving cognitive and emotional harm (Petric, 2022).

Three major definitions of gaslighting have been identified in the different streams of literature. Viewing from a sociological lens, according to Sweet (2019, p. 852), gaslighting is a “set of attempts to create a surreal social environment by making the other in an intimate relationship seem or feel crazy.” Gaslighting is also described as a “behavior in which one individual attempts to influence the judgment of a second individual by causing the latter to doubt the validity of his or her own judgment” (Calef and Weinshel, 1981, p. 52). In communications literature, gaslighting is defined as “a dysfunctional communication dynamic in which one interlocutor attempts to destabilize another’s sense of reality” (Graves and Spencer, 2022, p. 48). However, as mentioned earlier, the identified definitions lack the specificity to enable gaslighting to be distinguished from other constructs in the same nomological space and have not been operationalized. Thus, the authors of this study identify the key characteristics of gaslighting which is drawn from relevant research and supported by concept definition literature (Podsakoff et al., 2016) that not only provide the construct uniqueness, but also enhance its ability to be operationalized. Gaslighting involves- (1) Intentional/Unintentional tendency to harm the other party, and (2) Straightway rejection of concerns of others.

**What gaslighting is not:** Gaslighting should be differentiated from other harmful behaviors such as manipulation, lying, disagreement, guilt-tripping, stonewalling, ghosting, bullying, brainwashing, blackmailing, and name-calling. The definitions of these mentioned terms have been included in Table 1, however, it should be noted that not all of them are mutually exclusive and collectively exhaustive.

**TABLE 1:** Related terms and their definitions.

Construct	References	Definition
Manipulation	Susser et al., 2019, p. 6	“To covertly influence another person’s decision-making, by targeting and exploiting decision-making vulnerabilities.”
Lying	Isenberg, 1964, p. 466	“A statement made by one who does not believe it with the intention that someone else shall be led to believe it.”
Disagreement	Potter, 2013, p. 23	“A disagreement between A and B (where A and B are either individuals or groups of individuals) is a case where A accepts P and B rejects P.”
Guilt-tripping	Merriam-Webster, 2022	To try to manipulate the behavior of (someone) by causing feelings of guilt.
Stonewalling	Smithson and Venette, 2013, p. 399	“Uncooperative communication that strategically obstructs and delays the flow of information.”
Ghosting	Kay and Courtice, 2022, p. 406	“(i) is a relationship dissolution strategy, (ii) requires the ceasing of communication, (iii) occurs without an explanation, (iv) tends to occur suddenly rather than gradually, and (v) is enacted unilaterally by one of the partners in a relationship.”
Bullying	Escartín et al., 2013, p. 493	“Negative behaviors directed at organizational members or their work context that occur regularly and repeatedly over a period of time.”
Brainwashing	Scheflin and Opton, 1978, p. 87	“When a person has been compelled to believe subjectively a set of principles originally alien to him.”
Blackmailing	Blackmailing, 2022	The crime of demanding money from a person by threatening to tell somebody else a secret about them.
Name-calling	Steele, 1975, p. 361	“Conveying of a negative judgment, opinion, or evaluation of a person to that person.”

For instance, bullying has been defined as the perception of being at the receiving end of negative behaviors for a period of time and being unable to do anything to prevent it (Nielsen and Einarsen, 2012). Bullying and gaslighting may be gradual processes that an individual can be subjected to over a prolonged period of time. Bullying behaviors at work may be inflicted by those within and across organization hierarchies. However, gaslighting behaviors are often experienced where power differentials occur (Sweet, 2019), for example, between a supervisor and a subordinate. The Oxford Learners Dictionary defines brainwashing someone as forcing them to accept your ideas or beliefs either by repeating them or by preventing the other party to think clearly. Gaslighting involves distorting the other party’s sense of reality, thus, undermining other’s beliefs and thoughts is central to gaslighting behaviors.

## 2. Study development

The purpose of this work was to establish a clear definition of the concept and to build a reliable and valid measure of gaslighting that may contribute to the advancement of research in this area. The authors used a five-step process (Verreynne et al., 2016) to develop the scale for measuring gaslighting at work. In step one, a pool of potential items was generated on the basis of the characteristics of gaslighting defined above and through the use of secondary data. In study two, the items so generated were reviewed by a panel of eleven experts for face and content validation. In study three, exploratory factor analysis was conducted to identify the factor structure of the measure. In study four, confirmatory factor analysis was carried out to assess the dimensionality and reliability of the measure. In the final study, study five, a nomological study was conducted to test for criterion-related validity.

### 2.1. Step one: Item generation

As suggested by Hinkin (2016), preliminary items were developed on the basis of a theoretical foundation and definition of gaslighting. The study combined the deductive and inductive approaches to item generation. As also followed by Ahmad et al. (2020) and Perski et al. (2020), in the deductive approach, a thorough review of the existing literature on gaslighting and theoretically related variables such as workplace mistreatment, harmful leader behaviors and abusive relationships was used as a guide for developing items. On the other hand, in the inductive approach, related online blogs, Instagram posts, and tweets, were used to identify the characteristics of gaslighting and for developing further items. In total, 30 items were developed and subjected to a content validation study.

### 2.2. Step two: Content validation study

The initial 30 items developed from deductive and inductive approaches were tested for clarity of expression, understandability, and appropriateness subjected to expert review. A panel of eleven experts with academic/professional experience in the domain of organizational behavior and human resource management was presented with the authors’ definition of gaslighting and asked to review the pool of items. The panel was asked to critically evaluate each item and offer their suggestion on whether to retain, delete, reword or modify the item. The panel also assessed the items for clarity of expression, preciseness, redundancy, and readability level. The content validity was established when more than 50% of the experts agreed that the item was essential (Robinson, 2018). The suggested modifications were made thereto. At the end, 21 items were retained and considered further.

A small-scale pretest was carried out to identify any improvements for the items. To carry out the pretest, 35 working people (42% females, mean age = 27 years) participated in the study. The respondents shared their opinions on the shared items. All 21 potential scale items were retained in the study, however, the wording of 2 items was modified.

### 2.3. Step three: Item purification study (EFA)

A survey questionnaire was formed containing the final 21 items. Consistent with the development of similar scales like workplace mistreatment, workplace bullying, for developing GWQ, participants were asked to indicate how often they had mentioned experiences at work in the past 6 months on a scale of 1 to 5 from “Never” to “Always.” The 5-point scale to measure frequency has been chosen to keep it consistent with similar scales of workplace mistreatment and workplace bullying and minimize methodological variability (Cowie et al., 2002).

A total of 205 respondents from different organizations in service sector in India participated in the survey. Respondents ranged between 20 and 40 years of age (*M* = 30.96; SD = 8.032). A total of 132 respondents shared that they were unmarried, while 72 were married and 1 indicated that they were separated. Reported work experience included the range from 6 to 446 months (*M* = 70.32; SD = 85.619). A total of 113 respondents identified themselves as male, whereas, 90 respondents identified themselves as females, while 2 identified as third gender/non-binary.

The item-total correlations and inter-item correlations were calculated to ensure the internal consistency of the scale. In reference with Hiu et al. (2001) and Qian et al. (2007), items having <0.5 item-total correlations and <0.3 inter-item correlations were removed (Hair et al., 1998; Robinson, 2018). Four items exhibited item-total correlation <0.5, whereas three items exhibited inter-item correlation <0.3 (Hair et al., 1998). Thus, seven items, in total were omitted. To identify factor structure within the items, dimension reduction analysis (factor analysis) was conducted on 14 items.

Similar to previous studies (Gorbatov et al., 2021), exploratory factor analysis (EFA) using principal axis factoring with an Eigenvalue of more than 1.0 (Gharaibeh et al., 2019) was conducted and Promax rotation was specified to allow factors to be correlated (Hendrickson and White, 1964). The Kaiser–Meyer–Olkin value of 0.920 and Bartlett’s test of sphericity χ^2^ (66) = 1021.939, *p* < 0.05 indicated that the data set was suitable for the intended analysis and the correlation matrix was factorable. Thus, two more items were omitted.

Table 2 presents the items of the resulting scale, with their factor loadings and corrected item-total correlations.

**TABLE 2:** Final items, factor loadings, and corrected item-total correlations.

How often did you have these experiences at work in the past 6 months?			
	**EFA factor loadings**		Corrected item-total correlation
	Factor		
Item	1	2	
Your supervisor diverted the topic to project the fault onto you.	0.668		0.577
Your supervisor told you that you were “imagining” things.	0.548		0.575
Your supervisor passed degrading comments followed by rewards.	0.756		0.607
The words of your supervisor did not match with his/her actions.	0.583		0.627
Your supervisor denied the promises he/she made earlier.	0.675		0.608
Your supervisor undermined your complaints.	0.643		0.646
Your supervisor “twisted/misrepresented” things you said.	0.759		0.627
Your supervisor had unnecessary control over you.		0.721	0.666
Your supervisor made you your worst critic.		0.545	0.584
Your supervisor made you depend on him/her for making most of the decisions.		0.698	0.565
You felt emotionally drained at work because of your supervisor.		0.886	0.622
Your supervisor was very sweet to you one moment and very mean the other moment.		0.582	0.633
Extraction method: principal axis factoring.			
Rotation method: Promax with Kaiser normalization			

A total of 2 factors with Eigenvalues above 1.0 were extracted (Rejikumar and Asokan-Ajitha, 2022; Padhy and Hariharan, 2023). Table 2 reports the rotated component matrix with item/factor correlations for this two-factor analysis solution. Only the items that were extracted into factors are reported for greater clarity. A total of 12 items, forming two factors, were thereby identified. Each item had a minimum factor loading of 0.5, which is adequate (Hair et al., 2013).

After careful consideration and referring to the previous literature, the factors were interpreted and labeled as follows:

Factor 1: The items associated with Factor 1 (7 items) load on a construct that displays a tendency to oversimplify phenomena, have skeptical attitude toward the severity of a situation, and follow a casual approach as established by Pavelko and Myrick (2015). Thus, Factor 1 has been named Trivialization which refers to the undermining of subordinates’ perspectives, fears, and realities by the supervisor.

Factor 2: The items associated with Factor 2 (five items) load on a construct that elicits emotions of pain, suffering and torment as established by Robinson (2015). Thus, Factor 2 has been named Affliction, given its association with the negative emotions that a gaslighter (supervisor) can direct toward the target (subordinate).

The corrected item-to-total correlations varied from 0.577 to 0.666, and the Cronbach’s α of each dimension (α_*Trivialization*_ = 0.86, α_*Affliction*_ = 0.842) surpassed the accepted threshold of 0.80 (Clark and Watson, 1995), indicating the satisfactory reliability (internal consistency) of the scale. Therefore, the results confirmed that gaslighting at work construct consists of two factors. Additionally, the correlation coefficient between the two factors was 0.680. The high correlation between the factors endorsed that trivialization and affliction may comprise a second-order construct (Thompson, 2004; DeVellis, 2016). Subsequent confirmatory factor analysis aimed to further elucidate the dimensionality of the resultant scale. Therefore, the scale constituting 12 items was thenceforth subjected to confirmatory factor analysis.

### 2.4. Step four: Dimensionality and reliability study (CFA)

To verify the structure identified by EFA, Confirmatory factor analysis (CFA) was run. The items were readministered to a different sample of 216 participants to achieve statistical consensus for the factor structure. The respondents working in different organizations in service sector in India varied in age in the range of 20 to 54 years (*M* = 26.88 years, SD = 0.403). A total of 165 respondents identified themselves as males, 48 identified themselves as females, and 3 chose not to disclose their gender identity. The work experience of the respondents ranged from 6 months to 30 years (*M* = 36.47 months, SD = 47.045). The majority of the respondents were unmarried (81.8%).

A second-order construct was specified, having two dimensions-trivialization and affliction. The authors used multiple indices to evaluate the goodness of fit of the second-order model as shown in Table 3. These included chi-square/df = 1.244, comparative fit index (CFI) = 0.992, root mean square error of approximation (RMSEA) = 0.034, and the standardized root mean squared residual (SRMR) = 0.030. The acceptable fit was defined as chi-square/df less than 3, CFI values of 0.95 or greater, RMSEA values of 0.06 or less, and an SRMR of 0.10 or less (Hu and Bentler, 2009).

**TABLE 3:** Model fit indices of competing models.

Model	χ^2^	df	χ^2^/df	CFI	SRMR	RMSEA	NFI
One-factor model	100.755	52	1.938	0.969	0.042	0.066	0.939
1st order 2-factor model	106.058	53	2.001	0.967	0.039	0.068	0.936
Second order model	63.441	51	1.244	0.992	0.03	0.034	0.962

As previously shown in Table 2, all items loaded significantly on their respective dimensions with standardized loadings above 0.5. Composite reliability (CR) values for both the first-order dimensions (CR_*Trivialization*_ = 0.909, CR_*Affliction*_ = 0.876) and the second-order construct (CR_*Gaslighting*_ = 0.953) exceeded the cutoff value of 0.7 (Hair et al., 2013). Average variance extracted (AVE) values (AVE_*Trivialization*_ = 0.588, AVE_*Affliction*_ = 0.587, AVE_*Gaslighting*_ = 0.910) were above the 0.5 threshold (Fornell and Larcker, 1981).

Multiple tests were conducted to determine the discriminant validity between the dimensions of gaslighting. First, the 95% confidence intervals for estimated correlations between the two factors did not contain 1.0, indicating the divergence between the factors (Burnkrant and Page, 1982; Anderson and Gerbing, 1988). Second, in accordance with Bearden et al. (1989), the second-order model was compared against two competing models (i) in which each item loaded on a single factor (one-factor model), and (ii) first order two-factor model. The second-order model was significantly superior to the one-factor model and first order two-factor model (χ^2^ of second order model = 63.441, χ^2^ of one factor model = 100.755, χ^2^ of first order two-factor model = 106.058; *p* < 0.001). Third, the second order model has the lowest χ^2^/df, SRMR and RMSEA, and the highest CFI and NFI amongst the three models, which implies that the model fit indices of the second order model were superior fit indices when compared with the one-factor model and first order two-factor model as shown in the Table 3 (Hu and Bentler, 2009).

Overall, the results suggested that gaslighting is a second-order construct encompassing two first-order dimensions, trivialization and affliction. In addition, a reduced pool of 12 items (seven for trivialization, and five for affliction), was retained for the next study.

Further, it was important to assess the nomological validity of constructed gaslighting scale. Nomological validity examines whether or not empirical data supports a theoretical linkage between a construct and its antecedents or consequences (Cronbach and Meehl, 1955; MacKenzie et al., 2011). It was done by showing the relationship of gaslighting at work with other two constructs, one an antecedent and another consequence of gaslighting at work to provide evidence of nomological validity.

### 2.5. Step five: Nomological validity study

The nomological validity of the scale was assessed by checking whether the relationships with the constructs in the nomological space of abusive behaviors at work were significant.

To achieve the above-stated objectives, a three-phase study was conducted for data collection (Tripathi et al., 2022). The data collection was done as a part of a larger study. In the first phase (T1), data were collected on an antecedent of gaslighting at work. In the second phase (T2, T2 = T1 + 3 days); data were collected on the 12-item gaslighting at work scale and in the third phase (T3, T3 = T2 + 3 days) on a consequence of gaslighting at work. The relationships between the gaslighting at work scale and the theoretically related antecedent and consequence were then tested. The following section talks about the conceptualization of nomological validity for gaslighting at work and offers predictions about one specific antecedent and consequence.

#### 2.5.1. Hypothesis development

The authors chose role conflict as an antecedent and job satisfaction as a consequence for testing the nomological validity of GWQ. Role conflict is defined “in terms of the dimensions of congruence-incongruence or compatibility-incompatibility in the requirements of the role, where congruence or compatibility is judged relative to a set of standards or conditions which impinge upon role environments that would diminish an employee’s coping resources” (Rizzo et al., 1970, p. 155). Past research has shown that all parties involved in a bullying situation—victims, perpetrators, and bystanders—have reported role conflict to be a significant antecedent of bullying behaviors (Hauge et al., 2007). Role conflict is a role stressor that has been identified as one of the job stressors most consistently associated with complaints of workplace bullying (Reknes et al., 2019). Theoretically, Victim Precipitation Theory (Rock and Elias, 1987) helps understand the relationship and asserts that in certain situations, victims indulge in behaviors that ultimately cause their harm or injury. The conflicting role demands in the work environment lead to target’s frustration and perceiving of the situation as threatening, provokes negative behaviors (Topa et al., 2019) from others such as bullying and gaslighting. Therefore, it is proposed that:

H1: Role conflict at workplace is positively related to gaslighting at work.

Job satisfaction is a measure of total wellbeing and a key predictor of individual behavior (Clark, 1997). Job satisfaction has been defined as “a pleasurable or positive emotional state resulting from an appraisal of one’s job or job experiences” (Locke, 1976, p. 1300). The manner in which employees assess job satisfaction is contingent on their perceptions and evaluations of their job’s qualities and their physical and interpersonal work environment (Giorgi et al., 2015). Accordingly, the prevalent assumption is that exposure to abusive practises at work would cause employees to see their work environment as hostile and unpleasant (Bowling and Beehr, 2006) and gaslighting will thus negatively influence job satisfaction.

H2: Gaslighting at work is negatively related to job satisfaction of the employee.

**FIGURE 1:**
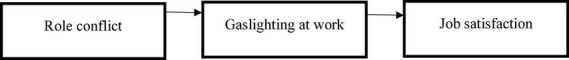
Conceptual model.

H1 and H2 can be represented in a conceptual model that establishes these three constructs- role conflict, gaslighting at work, and job satisfaction- in a nomological network and establishes a relationship as displayed in Figure 1.

#### 2.5.2. Procedure

In order to reduce the common method bias while testing the model, a time-lagged temporal design was used (Podsakoff et al., 2003). Respondents were US citizens aged 21 to 50 years, recruited through an online service named Prolific for participation in a three-phase online study. Data were collected in three phases T1, T2, and T3. There was a gap of 3 days between T1 and T2 and between T2 and T3 as suggested by Podsakoff et al. (2003). A temporal interval of 3 days, being of moderate duration for observing effects of psychological variables allows for the gradual unfolding of phenomena, while also mitigating the potential confounding influence of immediate effects.

In the first phase, T1, the role conflict as experienced by the subordinate was measured. In T2, data were collected on the 12-item gaslighting at work scale. Finally, in T3, the job satisfaction of the subordinate was captured. The final sample of the respondents who had completed all three instruments in T1, T2, and T3 was 258, comprising 131 male, 122 female, and 5 other respondents. Out of the respondents, 140 were unmarried, 110 married, 7 separated, and 1 widowed. The age of the respondents ranged from 21 to 62 years, Mean age = 36.5 years, SD = 9.93 years. The majority of the respondents (45.3%) had a bachelor’s degree, while 20.2% also had a master’s degree. The average number of months that the respondents have spent at their present organization was 65.6 months, SD = 36.6 months.

#### 2.5.3. Measures

Role conflict was measured using a six-item scale from Bowling et al. (2017). A sample item is “In my job, I often feel like different people are pulling me in different directions.” Cronbach’s alpha for the scale was 0.910. Gaslighting at work was assessed with the present authors’ 12-item measure of gaslighting at work. Cronbach’s alpha for the trivialization dimension of GWQ was 0.925 and for the affliction dimension of GWQ was 0.876. Job satisfaction was assessed using a three-item job satisfaction scale from the Michigan Organizational Assessment Questionnaire (Cammann et al., 1983). A sample item is “All in all, I am satisfied with my job.” Cronbach’s alpha for the scale was 0.921.

#### 2.5.4. Findings

As displayed in Table 4, the model fit indices of the model including role conflict as an antecedent and job satisfaction as a consequence of gaslighting at work, lie in the acceptable ranges (Hu and Bentler, 2009).

**TABLE 4:** Model fit indices for testing nomological validity.

Measure	Estimate	Threshold	Interpretation
CMIN	425.186	–	–
DF	181.000	–	–
CMIN/DF	2.349	Between 1 and 3	Excellent
CFI	0.937	>0.95	Acceptable
SRMR	0.076	<0.08	Excellent
RMSEA	0.072	<0.06	Acceptable
PClose	0.000	>0.05	Not estimated

Firstly, the convergent and discriminant validity of the constructs were established and later the hypotheses were tested, as also suggested by the two-step approach by Anderson and Gerbing (1988). Since the composite reliability (CR) >0.7, it is valid. As average variance extracted (AVE) >0.5, it has convergent validity. As maximum share variance (MSV) <AVE, it establishes discriminant validity. Table 5 provides the detailed statistics.

**TABLE 5:** Descriptive statistics and validity analysis.

	Mean	SD	CR	AVE	MSV	Gaslighting	Role conflict	Job satisfaction
Gaslighting	2.092	0.903	0.968	0.939	0.135	0.969		
Role conflict	3.953	1.436	0.901	0.604	0.135	0.367***	0.777	
Job satisfaction	4.301	1.318	0.924	0.802	0.119	-0.299***	-0.345***	0.896

***Significant at 0.001 level.

CR, composite reliability; AVE, average variance extracted; MSV, maximum shared variance; values on the diagonal represent the square root of AVE.

Controlling for gender, age, and educational level, role conflict was positively associated with gaslighting at work (β = 0.311, *p* < 0.01), i.e., when role conflict increases, gaslighting at work also increases. Similarly, controlling for gender, age, and educational level, gaslighting at work was negatively related to job satisfaction of the employee (β = −0.275, *p* < 0.01). It implies that gaslighting at work reduces the job satisfaction of employees. Therefore, empirical support is found for both Hypotheses 1 and 2. Thus, the relationship between role conflict and gaslighting behaviors, and gaslighting behaviors and job satisfaction has been established.

It was hypothesized that the prevalence of role conflict would lead to increased exposure to gaslighting behaviors and experiencing gaslighting at work would lead to reduced job satisfaction. The empirical evidence has been demonstrated through a time lagged study. Therefore, it is safe to say that the developed construct of gaslighting at work occupies a meaningful place in the nomological network.

## 3. Discussion

The study makes a significant contribution to the literature on harmful leader behaviors by conceptualizing and operationalizing gaslighting behavior in the workplace. Despite its harmful effects on individuals and organizations, gaslighting has received relatively little importance in the context of workplace. The study, thus aimed to address this lacuna in the literature.

The authors developed a 12-item Gaslighting at Work Questionnaire (GWQ) to measure gaslighting behaviors directed from a supervisor toward a subordinate. The analysis revealed that gaslighting is a second-order construct having two underlying dimensions: trivialization and affliction. Trivialization refers to the supervisor’s actions that undermine the subordinates’ perspectives, fears, and realities. On the other hand, affliction refers to the pain that the supervisor directs onto the subordinate. The authors thereafter, propose a refined definition of gaslighting as a negative workplace behavior wherein a person in position of power indulges in trivialization and affliction when dealing with subordinates. The proposed definition meets the requirements set forth by Granstrand and Holgersson (2020) as it satisfies an empirical and theoretical void, has enough precision, parsimony, and logical consistency without circularity, is operationalizable, qualifiable, typological, and usable for taxonomies, and is consistent syntactically and semantically with the prevalent conceptualizations of related topics.

**FIGURE 2:**
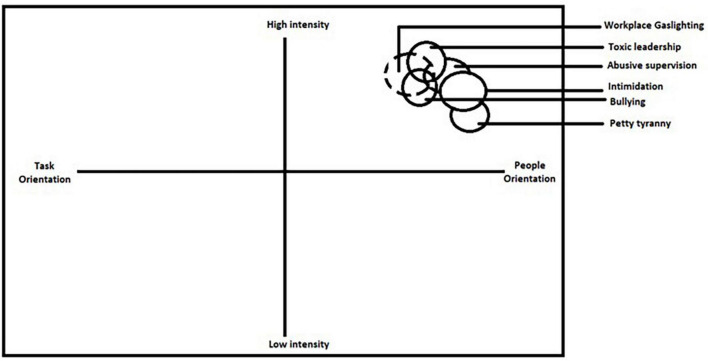
Gaslighting in the typology of harmful leader behaviors (Almeida et al., 2021).

Almeida et al. (2021) depicted harmful leader behaviors on a graph having two dimensions- intensity and orientation. In this depiction, gaslighting at work can be placed in Quadrant I as shown in Figure 2, which implies gaslighting at work has moderate to high intensity, and is people oriented. It also reveals some overlap of gaslighting at work with bullying, abusive supervision and toxic leadership, and provides valuable insights into the nature and consequences of gaslighting behavior. In conclusion, the proposed definition and operationalization of gaslighting at work in the form of GWQ provide useful tools for researchers and practitioners to identify and address gaslighting behavior in the workplace.

## 4. Contributions

Through this paper, the study makes three essential theoretical and empirical contributions to the literature on workplace abuse. First, to advance research on gaslighting, the study offers a clear and concise definition that encompasses two underlying dimensions: trivialization and affliction. Secondly, it adds to the literature on gaslighting by providing a reliable and valid scale to measure gaslighting at work. Furthermore, the study differentiates between gaslighting and other constructs in the same nomological sphere. Finally, the study is one of the initial attempts to draw attention and empirically demonstrate the importance of studying gaslighting in work settings. Specifically, the study provides empirical evidence that gaslighting is related to role conflict at the workplace and to job satisfaction of the employee.

## 5. Limitations and future research directions

There are certain limitations of the study that should be considered. Although the predictive validity of the developed scale has been established taking into account an antecedent and a consequence, it requires more investigation. Although control variables such as age, gender, educational level have been taken into consideration, other variables such as personality, tenure of the relationship may also affect the causal relationships in the study. Longitudinal studies showing various antecedents and consequences associated with gaslighting behaviors such as emotional labor (Pandey et al., 2018), job performance (Tett et al., 1991) knowledge hiding (Pandey et al., 2021), empowerment (Pandey, 2016) would be a valuable contribution toward a more comprehensive understanding of the concept of gaslighting at work. Possible mediators and moderators in the study of gaslighting in work settings are also encouraged. Gaslighting at work would lead to negative outcomes for the target employee, however, it is yet to be understood what all other factors may strengthen or weaken (moderate) this relationship, and may be able to better explain the linkage between gaslighting and unfavorable negative outcomes.

The scale is yet to be tested for other cultural settings as acceptance levels to abusive behaviors may differ from culture to culture (Power et al., 2013). The scale should be translated to other languages for using it in different cultures. Thus, the authors call for additional studies to verify the use of existing scale to the population beyond the current sample. The authors made sure to account for common method bias in the study and thus used a temporal gap to control for it. However, it should not be ignored that the study uses self-reported measures for the constructs under discussion. Thus, future studies using objective measures of the concepts under study are encouraged.

## Data availability statement

The raw data supporting the conclusions of this article will be made available by the authors, without undue reservation.

## Ethics statement

The studies involving human participants were reviewed and approved by the Institutional and/or National Research Committee and are in accordance with the 1964 Helsinki Declaration and its later amendments or comparable ethical standards. The patients/participants provided their written informed consent to participate in this study.

## Author contributions

Both authors listed have made a substantial, direct, and intellectual contribution to the work, and approved it for publication.
